# Adapting the One Health zoonotic disease prioritization tool for subnational One Health implementation through comparative analysis of disease severity and governance-driven approaches

**DOI:** 10.1016/j.onehlt.2026.101591

**Published:** 2026-09-21

**Authors:** Soawapak Hinjoy, Kittichai Unjit, Anyarat Thiptara, Wansane Toanan, Ariya Bunngamchairat, Pusana Rodsom, Sukunlaya Sirisukkarn, Sudarat Damrongwatanapokin, Serge Morand

**Affiliations:** a Department of Disease Control, Ministry of Public Health, 11000, Thailand; b Department of Livestock Development, Ministry of Agriculture and Cooperatives, 10900, Thailand; c Department of National Parks, Wildlife and Plant Conservation, Ministry of Natural Resources and Environment, 10900, Thailand; d Faculty of Public Health, Mahidol University, 10400, Thailand; e Department of Disease Control Foundation, 11000, Thailand; f IRL HealthDEEP CNRS, 10900, Thailand; g Faculty of Veterinary Technology, Kasetsart University, Bangkok 10900, Thailand; h Faculty of Tropical Medicine, Mahidol University, Bangkok 10900, Thailand

**Keywords:** One Health, Subnational health threats prioritization, Community engagement, Thailand

## Abstract

**Background:**

Zoonotic and environmentally driven health threats increasingly emerge at the human–animal–environment interface, highlighting the need for prevention-oriented and community-centered One Health approaches. This study adapted the OHZDP tool developed by the United States Centers for Disease Control and Prevention (U.S. CDC) for subnational use in Thailand, integrating environmental and social science perspectives and expanding community participation to ensure context-specific and operationally relevant prioritization.

**Methods:**

A comparative mixed-methods case study was conducted in two Thai provinces—Ubon Ratchathani and Nan—using multisectoral, four-day participatory workshops in 2025. Approximately seventy participants per province represented human, animal, environmental health, local governance, and civil society sectors. Health hazards were identified using an all-hazards approach. Six decision criteria were weighted through multisectoral consensus, and hazards were scored using a structured multi-criteria framework, complemented by qualitative deliberation to generate consensus-based prioritization.

**Results:**

Prioritization outcomes differed between provinces due to variations in criteria weighting. Ubon Ratchathani emphasized biomedical severity and cross-sectoral impact, prioritizing emerging infectious diseases, rabies, non-communicable diseases, and other high-mortality infections, reflecting a catastrophic risk preparedness profile. In contrast, Nan prioritized governance capacity and policy feasibility, resulting in rankings dominated by environmental and governance-dependent hazards, including PM2.5 air pollution, open waste burning, stray dog management, agricultural chemical exposure, and climate-related disasters.

**Conclusions:**

Community-led adaptation of prioritization tools strengthens subnational One Health action. Integrating governance, environmental, and social determinants into biomedical frameworks enhances multisectoral engagement, supports prevention-focused implementation, strengthens early warning systems, and builds community resilience against zoonotic disease emergence.

## Highlights

• Adapted the One Health zoonotic disease prioritization (OHZDP) tool for subnational health threats prioritization in Thailand.
• Participatory workshops integrated local knowledge with biomedical frameworks.
• Context-specific risks differ significantly from national-level aggregates.
• Community engagement enhances the legitimacy of zoonotic disease prioritization.
• The framework supports prevention-oriented implementation at the community level.

## 1 Introduction

Zoonotic diseases constitute 60–75% of emerging infectious diseases, reinforcing the need for prevention-oriented and community-driven strategies within One Health systems [1], [2], [3]. The Preventing Zoonotic Disease Emergence (PREZODE) initiative, a global framework aimed at preventing zoonotic disease emergence, promotes a shift toward anticipatory action through reducing spillover drivers, strengthening integrated surveillance, and embedding community participation in prevention pathways [4], [5]. Within this framework, the PREACTS (PREZODE in Action in the Global South) - ASAMCO project, funded by the Agence Française de Développement, has been implemented across in two countries in Southeast Asia, advancing multidisciplinary activities to implement innovative strategies to reduce and prevent zoonotic risk at the source (following the definition given by the One Health High-Level Expert Panel (OHHLEP) [6]), improve understanding of zoonotic risk factors and drivers, and strengthen early warning systems through government-science-society dialogue [7], [8]. These prevention-focused approaches emphasize community engagement, resource mobilization, and the co-production of knowledge, which have been shown to enhance early detection and achieve sustainable zoonotic risk reduction [9], [10], [11], [12]. By fostering local ownership of these initiatives, such frameworks not only improve surveillance but also build long-term resilience against emerging pathogens.

Operationalizing prevention in both PREZODE and ASAMCO contexts relies on zoonotic disease prioritization to guide targeted investments in surveillance and risk reduction [13]. Established frameworks such as the U.S. CDC One Health Zoonotic Disease Prioritization (OHZDP) tool — one of the One Health tools mapped in recent global inventories [14] — and multi-criteria decision analysis (MCDA) have been applied to build multisectoral consensus on zoonotic disease priorities across national and sub-national settings [15], [16]. However, studies highlight important limitations when applying these tools subnationally, where context-specific ecological risks, socio-cultural practices, and community vulnerabilities differ from national aggregates [17], [18], [19]. These limitations include insufficient representation of local stakeholders, minimal integration of environmental and social science inputs, and reliance on national-level datasets that may obscure local disease dynamics [20], [21].

To address these gaps, recent scholarship advocates for adapting existing prioritization tools through participatory, community-inclusive mechanisms that incorporate co-creation workshops, local ecological knowledge, and multi-sectoral weighting of criteria [18], [22]. Accordingly, this study modified the U.S. CDC OHZDP tool for application at the subnational level, embedding environmental and social science expertise and expanding community engagement to ensure contextually grounded, socially legitimate, and operationally relevant prioritization outcomes. This adaptation aligns with PREZODE-ASAMCO's emphasis on bottom-up prevention and resilience-building, and provides a robust foundation for community-led One Health action.

## 2 Materials and methods

### 2.1 Study design and setting

This study employed a comparative case study design to analyze the application and adaptation of a prioritization framework across two distinct provincial contexts in Thailand. Data were collected during two intensive four-day workshops conducted in late 2025: Ubon Ratchathani (October 2025) and Nan Province (November 2025). The study setting was selected to evaluate the framework's utility in translating national One Health policy into localized operational strategies, with particular attention to variations in ecological, socio-cultural, and governance contexts.

### 2.2 Adaptation of the CDC OHZDP tool

The standard OHZDP process is a structured, mixed-methods approach developed by the U.S. CDC to help a country or jurisdiction prioritize zoonotic diseases of greatest concern through multisectoral consensus [16]. Before the workshop, an initial list of candidate zoonotic diseases is compiled from national reportable-disease lists and a structured literature review, with input from the human, animal, and environmental health sectors. During a facilitated workshop, a panel of voting members representing these sectors collectively (i) defines a small set of prioritization criteria (typically five), (ii) develops one categorical question to measure each criterion, and (iii) ranks the criteria to derive their relative weights. Each candidate disease is then scored against every weighted criterion, and the scores are combined within the OHZDP Tool to generate a ranked priority list, which participants refine through group discussion and use to develop next steps and action plans [16]. Because voting members determine the criteria and weights relevant to their own setting, the tool is explicitly designed to be adaptable to local context, which provides the basis for the subnational adaptation described below.

In addition, the U.S. CDC OHZDP tool was modified to address subnational requirements through three key adaptations: (1) incorporation of environmental and social science criteria beyond biomedical metrics; (2) expansion of stakeholder categories to include community representatives, local ecological experts, and indigenous knowledge holders; and (3) integration of participatory weighting mechanisms allowing local actors to assign relative importance to prioritization criteria based on contextual relevance [16], [23], [24], [25].

### 2.3 Workshop structure and participants

#### 2.3.1 Multi-sectoral engagement and capacity building

The methodology followed a phased approach, beginning with national capacity building followed by provincial implementation:

• Facilitator Training: Twenty-four technical officers from the Department of Disease Control (DDC), Department of Livestock Development (DLD), and Department of National Parks (DNP) underwent training to master participatory prioritization techniques.
• Provincial Implementation: Each four-day workshop engaged 70 participants representing multiple sectors, including human health, animal health, environmental health, agriculture, wildlife conservation, local government, community organizations, and academic institutions.

#### 2.3.2 Pre-workshop data collection and threat identification

• Prior to the prioritization workshop, a bottom-up data collection phase was conducted to ensure local relevance. The central team distributed a standardized Excel template to all participating organizations in advance to facilitate the process. Each organization was tasked with listing health threats identified from the perspective of their specific sector and provinces
• Participants were required to provide a clear rationale for each proposed threat to ensure evidence-based selection.
• These submissions were returned to the central team, who performed data cleaning and thematic grouping. This process consolidated overlapping entries and prepared a finalized list of candidate health threats for formal voting and scoring during the workshop.

#### 2.3.3 Prioritization methodology: The analytic hierarchy process

**Step 1: Health Threat Identification and Data Cleaning**

Through a consensus-building exercise, participants regrouped and refined the list into a final set of prioritized health threats.

**Step 2: Consensus-Based Criteria Selection**

Participants collaboratively identified and defined six weighted criteria for assessing health threats:likelihood, severity, impact, vulnerability, control feasibility and policy support.

**Step 3: Criteria Weighting via Pairwise Comparison**

Facilitators guided participants through the Analytic Hierarchy Process (AHP). Using a standardized 1–9 scale, stakeholders conducted pairwise comparisons to determine the relative importance of each criterion.

**Step 4: Quantitative Risk Scoring**

Participants used guiding questions and a 1–5 scale to score all health threats against the established criteria. Scoring was oriented toward preparedness gaps: a score of 5 denoted the greatest gap (the lowest readiness to prevent, detect, or respond), so that higher scores identified the threats requiring the most urgent investment and support. The final prioritization was determined using a weighted-sum formula:

Total Weighted Score = Σ (Raw Score₍Criterion i₎ × Weight₍Criterion i₎).

Here, the Raw Score is the 1–5 value assigned to each health threat for a given criterion, and the Weight is the corresponding criterion weight obtained from the pairwise-comparison (AHP) exercise in Step 3, normalized so that the six criterion weights sum to 1. Each threat therefore received a single Total Weighted Score. To enable comparison across the full set of assessed threats, each threat's Total Weighted Score was then normalized to the sum of the Total Weighted Scores of all assessed threats and expressed as a percentage (Weighted Score %); threats were ranked in descending order of this value, with higher percentages denoting higher relative priority. The Weighted Score % reported in the prioritization tables (e.g., Table 1) was derived in this manner.

**Table 1:** Ubon ratchathani final threat prioritization (Top 10).

Rank	Threat/Disease	Weighted Score %	Strategic Justification
1	Emerging Infectious Diseases (Disease X)	5.3%	Represents the “unknown X" where preparedness is lowest across detection and response metrics.
2	Rabies	5.1%	High severity, requires multi-sectoral engagement, especially for stray animal management.
3	All Cancers (aggregate)	4.7%	Reflects the increasing national NCD burden.
4	Diabetes	4.3%	Key long-term health impact, aligned with national policy.
5	Impacts from Cross-border Armed Forces/Conflict	4.3%	Reflects the province's geopolitical position and forward-looking threat assessment.
6	Road Traffic Accidents	4.2%	High societal impact and mortality, are often overlooked in infectious disease prioritization.
7	Hypertension	4.2%	High prevalence NCD contributing to the long-term health burden.
8	Flooding	4.1%	Significant recurrent environmental threat impacting infrastructure and public health.
9	Melioidosis	4.1%	Regionally significant bacterial infection with high mortality.
10	Anthrax	4.0%	Significant zoonotic threat to livestock and human health.

Note: Weighted Score % denotes each threat's Total Weighted Score — calculated as the sum across the six criteria of (Raw Score [1], [2], [3], [4], [5] × criterion Weight), where weights were derived by pairwise comparison (AHP) and sum to 1 — expressed as a percentage of the summed Total Weighted Scores of all assessed threats; higher values indicate higher relative priority.

**Step 5: Synthesis and Deliberation Sessions**

Quantitative results were then refined through intensive, multisectoral deliberation sessions. These facilitated group reflections and plenary discussions were intended to harmonize differing sectoral perspectives and move beyond single-sector mandates. This combination of structured Multi-Criteria Decision Analysis (MCDA) and group dialogue was a critical step that allowed participants to qualitatively interpret the quantitative data and reach a final consensus on the strategic justifications for provincial ownership.

### 2.4 Data collection and analysis

Data were collected at the group level throughout both workshops and comprised: (i) the candidate health-threat lists and written rationales submitted by each participating organization on the standardized Excel templates during the pre-workshop phase; (ii) the six consensus prioritization criteria and their agreed definitions; (iii) the pairwise-comparison matrices and the resulting criterion weights generated through the Analytic Hierarchy Process; (iv) the 1–5 risk scores assigned to each health threat against each criterion, together with the resulting weighted-sum rankings; (v) facilitator field notes and records of the multisectoral deliberation sessions; and (vi) anonymous post-workshop feedback surveys. All data reflected group-level, consensus outputs of workshop activities and discussions; no individual-level personal information was collected. Quantitative data from scoring exercises were analyzed using weighted summation methods to generate disease priority rankings. Qualitative data from group discussions were analyzed thematically to identify patterns in stakeholder perspectives, decision-making processes, and contextual factors influencing prioritization outcomes. Comparative analysis between the two provincial workshops focused on identifying similarities and differences in disease priorities, criteria weighting, and stakeholder engagement patterns.

### 2.5 Ethical considerations

These workshops were conducted as programmatic One Health priority-setting and capacity-building activities led by the responsible Thai government agencies, rather than as research designed to collect individual-level human-participant data. Participants took part in their official capacity as representatives of their respective sectors and organizations, and their involvement was voluntary. The analysis used only aggregated outputs generated collectively during the workshops, including the consensus criteria, group weights and scores, and jointly agreed priorities. Post-workshop feedback was voluntary and anonymous and was analyzed only in aggregate. No names, personal identifiers, sensitive personal information, or individually attributable opinions were collected or reported; consequently, the findings cannot be linked to any individual participant. Because the activity did not constitute human-subjects research and no identifiable personal data were collected, formal research ethics committee approval was not required; the activity was nonetheless conducted in accordance with recognized ethical principles for public-health practice, including voluntary participation and confidentiality.

## 3 Results

### 3.1 Ubon Ratchathani: Prioritizing biomedical severity

In Ubon Ratchathani, the criteria weighting outcome was dominated by traditional health security metrics. The consensus weighting assigned the highest value to Severity (0.33), which accounted for direct quantitative health impacts such as case fatality, and Multi-sectoral Impact (0.32), which covered broader economic and social consequences. Collectively, these two criteria comprised approximately 65% of the total risk score, indicating a prioritization strategy focused on potential catastrophic outcomes.

The resulting prioritization matrix (Table 1) heavily featured emerging threats, high-mortality infectious diseases, and national non-communicable disease (NCD) burdens. Emerging Infectious Diseases (Disease X) ranked first, reflecting a strategy to mitigate risks where detection and response preparedness are lowest.

### 3.2 Nan Province: The governance-centric transformation

Nan Province initially followed a weighting scheme similar to Ubon, assigning severity a high weight of 0.39. However, during plenary deliberation, stakeholders posited that the magnitude of provincial threats was determined by local leadership capacity and policy enforcement rather than solely by biomedical severity. Consequently, the group executed a “Governance Determinant Thesis,” radically re-weighting the criteria: Severity was reduced to 0.03, while leadership/policy support was increased to 0.39.

This shift transformed the prioritization from a hospital-centered model to a provincial governance model. The final consensus-based ranking (Table 2) was dominated by recurrent environmental and persistent behavioral issues requiring strong policy coordination, with PM2.5 ranked first due to the necessity of multi-sectoral coordination.

**Table 2:** Nan province final threat prioritization (Top 8).

Rank	Consensus-Based Final Top 8 Hazards	Primary Strategic Justification
1	PM 2.5	Requires multi-sectoral (forestry, environment, agriculture) coordination and leadership (0.39 weight).
2	Severe Acute Respiratory Diseases	Linked to environmental factors (haze) and high transmission, requiring governance alignment.
3	Stray dogs & rabies	Primarily a governance issue concerning municipal ordinances, budget, and community acceptance.
4	Agricultural chemical hazards	Driven by farming practices, limited supervision, and local enforcement capacity.
5	Tuberculosis + XDR-TB	Severity combined with resource-intensive containment efforts.
6	Floods/landslides/ water-related disasters	Management effectiveness depends on provincial disaster command structures.
7	Waste management and burning	A fundamental governance and behavioral challenge in the province.
8	*Streptococcus suis*	A significant zoonotic threat influenced by consumers' behavior.

### 3.3 Comparative analysis of criteria weighting and strategic drivers

The divergence in strategies was quantified by direct comparison of criteria weights (Table 3). This comparison reveals that Ubon Ratchathani focused on biomedical consequences (severity + impact), whereas Nan Province focused on governance feasibility (leadership/policy).

**Table 3:** Criteria weighting comparison across provinces.

Criterion	Ubon Ratchathani	Nan (Initial)	Nan (Final Governance Weight)	Strategic Focus
Severity/Health Impact	0.33	0.39	0.03	Biomedical Consequence
Leadership/Policy Support	0.08	0.03	0.39	Governance/Feasibility
Magnitude/Transmission	0.14	0.31	0.31	Epidemiological Spread
Treatment & Prevention Capacity	0.12⁎	0.12	0.12	System Readiness
Economic/Social Impact	0.32	0.16	0.16	Broader Consequences

⁎ Note: Ubon Ratchathani split this into Detection (0.06) and Response (0.06).

This weighting difference resulted in significantly disparate final priority lists (Table 4). Ubon Ratchathani's inclusion of NCDs and geopolitical threats reflects a systemic assessment of national public health challenges, while Nan's list emphasizes environmental stressors and municipal governance challenges.

**Table 4:** Comparative top hazards: ubon ratchathani vs. Nan.

Rank	Ubon Ratchathani (Severity Focus)	Nan Province (Governance Focus)	Primary Strategic Driver
1	Emerging Infectious Diseases (Disease X)	PM 2.5	Preparedness vs. Environmental Governance
2	Rabies	Severe Acute Respiratory Diseases	Severity vs. Governance Enforcement
3	All Cancers (aggregate)	Stray dogs & rabies	Chronic NCD Burden vs. Municipal Governance
4	Diabetes	Agricultural chemical hazards	Chronic NCD Burden vs. Agricultural Policy
5	Impacts from Armed Forces/Conflict	Tuberculosis + XDR-TB	Geopolitics vs. Severity
6	Road Traffic Accidents	Floods/landslides	Societal Impact vs. Disaster Command
7	Hypertension	Waste management and burning	Chronic NCD Burden vs. Municipal Governance
8	Flooding	*Streptococcus suis*	Environmental vs. Zoonotic Interface

Ubon Ratchathani's prioritization reflected a systemic assessment of public health challenges and response infrastructure. In contrast, Nan's final list is dominated by environmental stressors (PM2.5, floods) and behavioral/municipal governance challenges (stray dogs, chemical hazards, waste burning). Identifying PM2.5 as the top priority hazard validates the heavyweight assigned to governance, reflecting the consensus that successful mitigation requires complex, multi-sectoral leadership rather than a standalone health response. The strategic difference confirms that the MCDA criteria weighting was the most important variable defining whether the prioritization output functions as a catastrophic risk map or an operational feasibility plan.

## 4 Discussion

This study demonstrates that adapting national prioritization frameworks for subnational application requires fundamental methodological shifts beyond simple scaling. The modified OHZDP tool successfully addressed key limitations identified in previous studies [17], [19] by incorporating environmental and social science perspectives, expanding stakeholder participation, and enabling context-sensitive criteria weighting. These adaptations enhanced the framework's capacity to capture local disease dynamics, community vulnerabilities, and socio-ecological complexities that national-level approaches often overlook. Our study's expansion of the prioritization scope to include environmental hazards (PM2.5, flooding) and non-communicable diseases (diabetes, hypertension) reflects this subnational reality, where communities experience health threats as interconnected challenges rather than discrete disease categories.

The comparative analysis revealed a productive tension between biomedical and governance-centric approaches. This tension is most evident in the differing priorities of the public health and environment sectors, where the need for technical disease control (biomedical) must be balanced against the complexities of land management and cross-sectoral policy (governance). Ubon Ratchathani's emphasis on epidemiological severity and economic impact aligns with traditional biomedical paradigms. This approach prioritizes quantifiable health metrics and measurable economic outcomes over qualitative factors [26], [27]. In contrast, Nan Province's attention to community vulnerability, environmental sustainability, and cultural significance exemplifies governance-centric approaches that foreground social equity, environmental justice, and participatory decision-making [28], [29]. Both approaches offer valuable insights; however, achieving optimal prioritization requires a dynamic integration that can only be realized through the development of next-generation One Health tools. As emphasized by a recent One Health scoping review of prioritization tools, there is a critical need for frameworks that move beyond biomedical dominance to more robustly integrate environmental and governance indicators as core components of the decision-making process [19].

The study's emphasis on community engagement extends beyond procedural participation to epistemological inclusion—recognizing community members as legitimate knowledge producers whose experiential insights are essential for understanding zoonotic risk [30], [31]. The modification implemented in this study—allowing participants to reweight criteria to reflect governance feasibility—marks a significant shift from pathogen-centric to systems-oriented prioritization. This approach directly operationalizes the OHHLEP definition of upstream prevention by targeting the drivers of zoonotic emergence. It recognizes that health threats arise from modifiable risk factors within the ecosystem; by prioritizing governance-ready interventions, we can address these drivers before they progress to spillover or disease states. For example, agricultural chemical exposure, inadequate waste management, and air quality degradation (all prioritized in Nan) are not diseases per se, but rather risk conditions that require multisectoral risk mitigation strategies involving agriculture, environment, and municipal governance sectors. The successful integration of community knowledge in both workshops demonstrates the feasibility and value of epistemological pluralism in One Health practice.

The governance-determinant thesis adopted in Nan Province illustrates the practical implications of this modification. By elevating leadership and policy support to the highest-weighted criterion (0.39), participants effectively transformed the prioritization from a hospital-centered preparedness plan into a provincial development agenda. The resulting priority list—dominated by PM2.5, stray dog management, agricultural chemical safety, and waste management—reflects issues that require sustained political commitment, budget allocation, and coordination across municipal, environmental, and agricultural authorities. These priorities would likely have been marginalized in a severity-weighted framework despite their profound impacts on population health and their dependence on multisectoral governance for effective mitigation.

This mixed-methods approach addresses a fundamental challenge in complex systems assessment. While purely quantitative methods provide transparency and comparability, they risk fragmenting understanding; conversely, qualitative methods capture essential context and stakeholder perspectives but often lack systematic structure [15]. Osman et al. [1] demonstrated that the OHZDP process in Somalia generated more than simple priority rankings. Through facilitated cross-sectoral dialogue, the process also generated joint strategic actions and strengthen surveillance linkages. These examples, together with our findings from Thailand, suggest that the deliberative component of mixed methods is not merely a validation step but an essential mechanism for translating technical analysis into collective action.

This study's findings support the argument that One Health prioritization tools must be sufficiently flexible to accommodate diverse entry points for different sectors by demonstrating how participatory prioritization can identify upstream drivers of zoonotic risk and mobilize community capacity for early detection and response [4], [32], and by better inclusion of the environment [OHHLEP]. By engaging communities as active partners rather than passive beneficiaries, the adapted framework enhances the sustainability and legitimacy of One Health interventions. This shift from reactive disease control to proactive prevention requires sustained investment in participatory processes, capacity building, and equitable resource distribution [33]. Participatory epidemiology literature emphasizes that qualitative methods create essential space for diverse voices, particularly those representing community perspectives, gendered vulnerabilities, and practical implementation constraints that quantitative indicators may not capture [11].

Several limitations warrant consideration. First, the study focused on two provinces within a single country, limiting generalizability to other contexts. Second, the four-day workshop format, while intensive, captures priorities at a single point in time and may not reflect how stakeholder participation and agreed priorities shift over subsequent months or years—for example, as leadership changes, new threats emerge, or resource conditions evolve. To address this limitation, future applications could incorporate longitudinal follow-up mechanisms—such as periodic re-scoring exercises, digital dashboards, or annual review cycles—to iteratively update priorities and strengthen the adaptive use of One Health decision-support tools. Third, resource constraints limited the depth of post-workshop follow-up to assess implementation outcomes. Future research should examine the long-term sustainability of participatory prioritization processes, assess implementation fidelity and impact, and explore scalability across diverse governance contexts. Additionally, comparative studies across countries with varying One Health maturity levels would enhance understanding of contextual factors influencing prioritization effectiveness.

## 5 Conclusion

This study demonstrates that subnational One Health prioritization, when conducted through participatory mixed-methods approaches with flexible tools, can serve as a powerful foundation for multisectoral partnership and prevention-oriented action. The findings challenge the assumption that single prioritization frameworks should be uniformly applied across contexts, instead supporting adaptive methodologies that allow local stakeholders to define the criteria and weights that reflect their operational realities. By modifying biomedical-centric tools to accommodate governance and social determinants, and by integrating quantitative structure with qualitative deliberation, this approach creates space for diverse sectors to identify their roles in One Health systems. These lessons are particularly relevant for operationalizing the PREZODE PREACTS-ASAMCO vision of community-centered, prevention-focused One Health that addresses the upstream drivers of health threats rather than solely preparing to respond to disease emergence.

## Declaration of generative AI and AI-assisted technologies in the manuscript preparation process

During the preparation of this work the authors used ChatGPT by OpenAI and Claude in order to improve language. After using this tool/service, the authors reviewed and edited the content as needed and take full responsibility for the content of the published article.

## CRediT authorship contribution statement

**Soawapak Hinjoy:** Writing – review & editing, Writing – original draft, Validation, Methodology, Formal analysis, Data curation, Conceptualization. **Kittichai Unjit:** Writing – review & editing, Validation, Investigation, Formal analysis, Conceptualization. **Anyarat Thiptara:** Writing – review & editing, Methodology, Investigation, Formal analysis, Conceptualization. **Wansane Toanan:** Methodology, Investigation, Formal analysis, Data curation. **Ariya Bunngamchairat:** Supervision, Methodology, Investigation, Formal analysis, Conceptualization. **Pusana Rodsom:** Validation, Methodology, Investigation. **Sukunlaya Sirisukkarn:** Writing – review & editing, Project administration, Investigation. **Sudarat Damrongwatanapokin:** Writing – review & editing, Supervision, Conceptualization. **Serge Morand:** Writing – review & editing, Supervision, Formal analysis, Conceptualization.

## Funding

This work has received support from the ASAMCO project, funded by the 10.13039/501100011061French Development Agency (AFD) as part of PREACTS (PREZODE in action in the global South) program).

## Declaration of competing interest

The authors declare that they have no known competing financial interests or personal relationships that could have appeared to influence the work reported in this paper.

## Data Availability

Data will be made available.
